# Neuroprotective potential of eugenol against acrylamide-induced brain toxicity by regulating Nrf2/NQO1/HO-1 and NLRP3/NF-κB/IL-1β signaling cascades

**DOI:** 10.1038/s41598-025-34650-8

**Published:** 2026-01-23

**Authors:** Sara M. Baraka, Yosra A. Hussien, Omar A. Ahmed-Farid, Azza Hassan, Dalia O. Saleh

**Affiliations:** 1 Chemistry of Natural Compounds Department, National Research Centre, Giza, 12622 Egypt; 2 Pharmacology Department, National Research Centre, Giza, 12622 Egypt; 3 Physiology Department, National Organization for Drug Control and Research, Giza, Egypt; 4 Pathology Department, Faculty of Veterinary Medicine, Cairo University, Giza, Egypt

**Keywords:** Eugenol, Acrylamide, Neurotoxicity, Nrf2/HO-1 pathway, NLRP3 inflammasome, Apoptosis, Biochemistry, Drug discovery, Neurology, Neuroscience

## Abstract

Acrylamide (ACR), a common environmental and dietary neurotoxicant, exerts profound deleterious effects on the central nervous system by triggering oxidative stress, neuroinflammation, apoptosis, and motor impairments. Eugenol (EU), a natural phenolic compound known for its antioxidant and anti-inflammatory properties, was evaluated for its neuroprotective efficacy in ACR-induced brain toxicity in rats. Male Wistar rats were orally administered ACR (20 mg/kg/day) for four weeks to induce neurotoxicity, with concurrent administration of EU at two doses (50 and 100 mg/kg/day). Behavioral assessments, including foot splay, gait score, and rotarod performance, were conducted to evaluate motor coordination and neuromuscular integrity. Biochemical analyses revealed that ACR significantly elevated markers of oxidative and nitrosative stress, suppressed antioxidant defense mechanisms. Furthermore, ACR induced significant upregulation of pro-inflammatory mediators (NLRP3, p-NF-κB, IL-1β), as well as apoptosis markers such as caspase-3, alongside prominent histopathological alterations and astrocyte activation (evidenced by increased GFAP expression). Treatment with EU resulted in a dose-dependent amelioration of these neurotoxic effects. Notably, EU restored motor function, attenuated oxidative/nitrosative damage, and reactivated the Nrf2/NQO1/HO-1 antioxidant pathway. Simultaneously, it significantly downregulated the expression of NLRP3, p-NF-κB, and IL-1β, indicating strong anti-inflammatory action. Histological analysis confirmed preservation of neuronal architecture, while immunohistochemistry showed reduced caspase-3 and GFAP expression in EU-treated groups. These findings suggest that EU exerts potent neuroprotective effects against ACR-induced brain toxicity, primarily through modulation of redox balance, suppression of neuroinflammation, and inhibition of apoptotic cell death, via targeting both the Nrf2-mediated antioxidant system and the NLRP3/NF-κB/IL-1β inflammatory cascade.

## Introduction

Acrylamide (ACR) is a water-soluble α,β-unsaturated carbonyl compound extensively used in industrial processes such as paper manufacturing, dye production, and wastewater treatment^1^. It is also formed in carbohydrate-rich foods during high-temperature cooking via the Maillard reaction. Growing evidence from both epidemiological and experimental studies has established ACR as a potent neurotoxic agent, with the nervous system being a primary target of its toxic effects^2^. In both humans and experimental animals, chronic exposure to ACR results in progressive neurotoxicity, with clinical manifestations including skeletal muscle weakness, ataxia, foot splay, and impaired motor coordination, symptoms that are strongly correlated with cerebellar dysfunction and neuronal degeneration^3^.

Mechanistically, ACR-induced neurotoxicity has been linked to several interrelated pathways, notably oxidative stress, neuroinflammation, and neuronal apoptosis^4^. ACR and its reactive metabolite glycidamide interact with cellular macromolecules, leading to glutathione (GSH) depletion, lipid peroxidation, and increased production of reactive oxygen and nitrogen species^5,6^. This causes oxidative damage and impairment of endogenous antioxidant defenses, notably through suppression of the nuclear factor erythroid 2–related factor 2 (Nrf2) signaling pathway, which plays a critical role in cellular redox homeostasis by inducing cytoprotective genes such as HO-1 and NQO-1^7,8^.

Moreover, the activation of the NLRP3 inflammasome and the NF-κB signaling pathway further exacerbates ACR-induced neuroinflammation. This inflammatory cascade is characterized by increased expression of pro-inflammatory cytokines such as IL-1β and TNF-α, which can potentiate neuronal injury and glial activation^9^. Importantly, recent studies have elucidated a crosstalk between the Nrf2 and NLRP3 pathways, whereby the suppression of Nrf2 exacerbates inflammasome activation, suggesting a tightly regulated balance between oxidative and inflammatory signaling in ACR-induced neuropathology^10,11^. Concurrently, astrocytic activation (astrogliosis), characterized by overexpression of glial fibrillary acidic protein (GFAP), represents another hallmark of ACR-induced neurodegeneration^12^.

Given these multifaceted pathogenic mechanisms, there is a growing interest in identifying natural compounds with pleiotropic protective effects against ACR-induced neurotoxicity. Eugenol (EU), a phenolic compound derived from clove oil, has garnered attention for its potent antioxidant, anti-inflammatory, and neuroprotective properties^13^. Previous studies have demonstrated that EU can activate the Nrf2 pathway, scavenge free radicals, and inhibit inflammatory mediators such as NF-κB and NLRP3^14–16^. However, its therapeutic efficacy against ACR-induced neurotoxicity remains underexplored.

Therefore, the present study was designed to comprehensively investigate the neuroprotective potential of EU in a well-established rat model of ACR-induced brain toxicity. The objective is to assess whether EU can mitigate ACR-associated behavioral deficits, oxidative and nitrosative stress, neuroinflammation, apoptosis, and histopathological changes. Particular emphasis is placed on exploring the molecular mechanisms underlying these effects, focusing on the possible activation of the Nrf2/NQO1/HO-1 antioxidant defense pathway and the suppression of the NLRP3/NF-κB/IL-1β inflammatory axis.

## Material and methods

### Drugs and chemicals

Acrylamide (ACR) was sourced from Sigma-Aldrich (A3553), St. Louis, MD, USA, and was freshly prepared in distilled water with a concentration of 8 mg/mL. MD, USA. It was formulated in 1 mL of olive oil to achieve a concentration of (50 mg/mL and 100 mg/mL) for preparing the doses of 50 mg/kg and 100 mg/g, respectively. The ACR and eugenol were orally administered. The remaining substances were all of the highest analytical quality available. Rat ELISA kits were utilized for phosphorylated nuclear factor kappa-light-chain-enhancer of activated B cells p65 subunit (p-NF-κB p65) (SBRS1911, PharmaGenie ELISA kits, AssayGenie, Ireland), interleukin-1 beta (IL-1β) (E0119Ra, Bioassay Technology Laboratory, Zhejiang, China), NOD-like receptor protein 3 (NLRP3) (ER1965, Fine Test®, Wuhan, China), heme oxygenase-1 (HO-1) (ER1041, Fine Test®, Wuhan, China), NAD(P)H quinone dehydrogenase 1 (NQO1) (A87472, Antibodies.com LLC, MO 63,132, USA), and nuclear nuclear factor erythroid 2–related factor 2 (nuclear Nrf2) (RD-NF2L2-Ra, www.reddotbiotech.com, Kelowna, BC V1W 4V3, Canada).

### Experimental animals and ethical approval

This study utilized male Wistar Albino rats (n = 24) aged four months and weighing 180–220 g. The animals were obtained from the animal house of the National Research Centre (Giza, Egypt). Throughout the study, the rats were kept in suitable conditions within a room that had 12 h of daylight. They were provided with appropriate cages, fed standard rat pellet chow sourced from the animal house at the National Research Centre, and given free access to water through dropper-tipped bottles.

The study protocol adhered to the ethical guidelines set forth by the approval from the Medical Research Ethics Committee (MREC), National Research Centre (NRC), Cairo, Egypt (Approval No. 24410112022), and in accordance with the ARRIVE guidelines.

Following the instructions outlined in the 8th edition of IACUC for pain management for laboratory animals^17^, all necessary precautions have been fulfilled to prevent and/or alleviate the animal distress by applying strategists such as analgesics if possible, and humane endpoint if required. During the experiment, the animals’ behavior, mobility, respiratory rate, and postural shifts, as well as the body weight, and body temperature were checked out once a day by a clinical veterinarian and/or a well-trained investigator. Before the planned euthanasia, no mortality cases were recorded in the studied groups.

### Protocol of the in vivo study

Twenty-four male Wistar rats were randomly divided into four equal groups: a control group that received vehicles orally (1 mL olive oil/kg and 2.5 mL distilled water); a model group (ACR) that was given oral ACR (20 mg/kg bw) and olive oil (1 mL/kg) daily for 4 consecutive weeks; and two eugenol-treated groups (EU50 + ACR and EU100 + ACR) that received eugenol via oral route (EU; 50 or 100 mg/kg bw/day, respectively) alongside daily ACR doses for 28 days, with EU administered before ACR exposure at a one-hour interval to allow sufficient time for its absorption and systemic distribution^18^. The doses of ACR and eugenol were chosen based on a previous work^16^.

Twenty-four hours post administration of the final drug dose; the rats were sacrificed by decapitation under ketamine–xylazine anesthesia (50–5 mg/kg, i.p)^19^. At autopsy, the brain was carefully gathered from each rat. Some brain tissue portions were stored at − 80 °C for subsequent biochemical analyses, while other specimens were fixed in 10% formalin for histopathological and immunohistochemical studies.

Animals were randomly assigned to treatment groups using a simple randomization method to minimize selection bias. Both the investigators responsible for drug administration and those performing biochemical, histological, and immunohistochemical analyses were blinded to group allocation throughout the experimental and analytical procedures.

### Behavioral assessments

#### Gait score

For each rat per group, the gait score was documented before the experiment commenced and then weekly over a four-week period. The movement of each rat was observed for duration of 3 min, and a score was assigned based on the following standards that set forth by LoPachin et al.^20^: normal gait given 1; minor foot splay and slight ataxia in hind limbs given 2; score 3 assigned for rats showed reasonable foot splay distance and moderate hind limb weakness during walking; and score 4 given for rats with severe hind limb dimness recognized by dragging hind limbs and incompetence to rear.

#### Landing hind limb foot splay distance

In accordance with the procedure described by LoPachin et al.^20^, measurements were taken of the distance of foot splay in the hind limbs. The rat’s hind foot was dipped in ink, and the rat was dropped from a height of 20 cm onto white paper. The distance between the centers of the rat’s hind heels was measured weekly throughout the experiment.

#### Rotarod

Motor coordination and balance were assessed using a rotarod apparatus, following the method described by Bohlen et al.^21^. Rats were placed on a rotating rod (diameter 7 cm) set to accelerate gradually from 4 to 40 revolutions per minute over a period of 300 s. The latency to fall (time each rat remained on the rod without falling) was recorded. Each rat underwent three trials with a 15-min rest between trials, and the mean latency was calculated.

### Biochemical investigations

#### Evaluation of brain tissue oxidative and nitrosative stress biomarkers

The levels of reduced and oxidized glutathione (GSH, and GSSG respectively) were analyzed using a High-Performance Liquid Chromatography (HPLC) system from Agilent HP 1200 series, made in the USA. The system included a column oven, a quaternary pump, a UV variable wavelength detector, and a Rheodyne injector with a 20 μL loop. The analytical column employed was a μBondapak C18 column, featuring a particle size of 10 μm and a pore size of 125 Å, with dimensions of 3.9 mm × 300 mm (Catalog Number: WAT027324). The GSH and GSSG levels were quantified using reference standards from Sigma Chemical Co., and the results are expressed in μmol/g tissue^22^.

Using an HPLC system with a Supelcosil C18 column (particle size: 5 μm, pore size: 120 Å, dimensions: 250 × 4.6 mm, Catalog Number: 58298), the levels of malondialdehyde (MDA) were measured. To create a 1 mM stock solution for the MDA standard, 25 μL of 1,1,3,3-tetraethoxypropane (TEP) was dissolved in 100 mL of water. By hydrolyzing 1 mL of the TEP stock solution in 50 mL of 1% sulfuric acid and incubating it for 2 h at room temperature, a working standard was prepared. It was further diluted with 1% sulfuric acid to reach a concentration of 20 nmol/mL TEP, which was then adjusted to a final concentration of 1.25 nmol/mL for total MDA estimation^23^.

The level of nitric oxide (NO), particularly nitrate/nitrite, was measured using the Agilent HP 1200 series HPLC system from the USA, according to the specified method. An anion exchange PRP-X100 Hamilton column with dimensions of mm and a particle size of 10 μm was employed. The mobile phase was made up of 0.1 M NaCl and methanol mixed in a volume ratio of 45:55. The flow rate was maintained at 2 mL/min, and detection occurred at a wavelength of 230 nm. This configuration enabled the examination of NO levels as nitrate/nitrite^24^, with concentration assessed by comparing the chromatogram to the standard provided by Sigma Aldrich.

#### Assessment of brain p-NF-κBp65, IL-1β, NLRP3, HO-1, and NQO1 and nuclear Nrf2 levels

Using a polytron homogenizer, a 10% brain tissue homogenate was created in 0.05 M PBS (pH 7). The homogenate produced was centrifuged at 10,000 rpm for 20 min, which removed cell debris, unbroken cells, nuclei, erythrocytes, and mitochondria. The resulting supernatant (cytosolic fraction) was carefully isolated for the determination of p-NF-κBp65, IL-1β, NLRP3, HO-1, and NQO1 levels using rat ELISA kits.

On the other hand, the remaining pellet was immersed in 500 μL Nuclear Lysis Buffer to produce the nuclear fraction. The suspension was subjected to spinning in a cooling centrifuge for 10 min at 14,000 rpm. The resulting supernatant (nuclear fraction) was gathered for the quantitative assessment of nuclear Nrf2 content using a rat ELISA kit. The protein concentration for each sample was assessed using a kit supplied from Genei, Bangalore.

### Histopathological examination

The brains from all groups were removed and fixed in neutral buffered formalin (10%) for 48 h. The tissues were processed and embedded in paraffin blocks. After that, the tissues were cut into 4µm-thick sections and stained with hematoxylin and eosin for light microscopical examination. For assessment of neurotoxicity, the number of degenerate neurons in the cerebral cortex and hippocampus, as well as the degenerate purkinje cells, were counted in five random high-power fields (40X), as reported by Radad et al.^25^.

### Immunohistochemical analysis

Caspase-3 immunohistochemical staining for assessment of apoptosis in the brains was carried out. Additionally, GFAP immunohistochemical staining is performed to demonstrate the reactive astrocytes in the cerebral cortex of all groups. Briefly, the paraffin-embedded tissues were sectioned, dewaxed, and rehydrated in xylene. Antigen retrieval was then carried out in a microwave. After that, the sections were treated with 3% hydrogen peroxide to inhibit endogenous peroxidase activity. The tissues were then incubated with rabbit monoclonal anti-caspase-3 [EPR18297] (ab184787) (Abcam), and rabbit monoclonal anti-GFAP [EPR1034Y] (ab68428) as primary antibodies. Following washing with phosphate buffered saline and secondary antibody incubation, diaminobenzidine was added to stain the sections. The sections were then counterstained with hematoxylin. Caspase-3 was semi-quantitively assessed in ten random high-power fields (40X), according to the percentage of positive cells, in which scale 0 = no staining, scale 1 = denotes staining in 25%, scale 2 = 25%–50%, scale 3 = 51%–70%, and scale 4 = 70%. On the other hand, for assessment of GFAP immune staining, the GFAP + cells were counted in five random high-power fields (40X).

### Statistical analysis

Data are presented as mean ± SEM. The Shapiro–Wilk’s test was employed to check for normality at *p* > 0.05 (Shapiro and Wilk, 1965). For multiple comparisons, a one-way analysis of variance (ANOVA) followed by Tukey’s post hoc test was used, considering values significant at *p* < 0.05 or 0.001. All statistical analyses were conducted using the GraphPad Prism (version 9.0, GraphPad Software, San Diego, California, USA).

## Results

### Effect of eugenol on behavioral changes in ACR-induced brain toxicity in rats

**Fig. 1:**
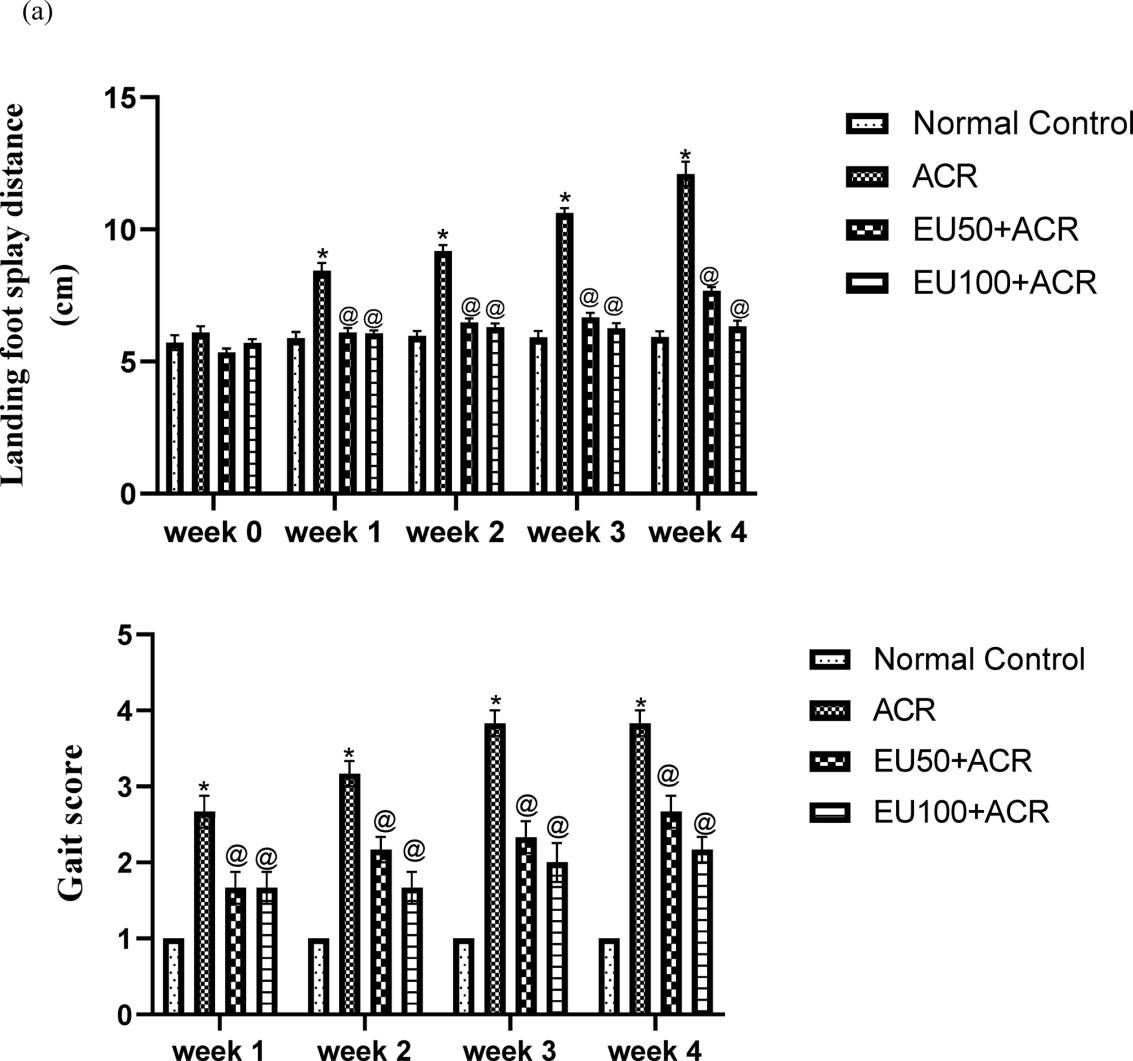
Effects of eugenol on landing foot splay distance (**a**) and gait assessment score (**b**) in rats with ACR-induced brain toxicity. The data are presented as mean ± SEM (n = 6). “*” represents a significance difference vs normal control group at *P* value < 0.05, “^@^” represents a significance difference vs ACR group at *P* value < 0.05.

The behavioral assessment revealed that ACR administration led to significant motor disturbances in rats, as indicated by a progressive increase in landing hind limb foot splay distance (Fig. 1a) and gait score (Fig. 1b) across the 4-week experimental period. Specifically, administration of ACR induced pronounced behavioral disturbances in rats, as evidenced by a significant increase in landing foot splay distance and elevated gait scores. Such motor impairments reflect the neurotoxic consequences of ACR, which can negatively impact both central and peripheral neuromotor functions.

Remarkably, co-administration of EU exerted a protective effect, significantly ameliorating the observed behavioral deficits in a dose-dependent fashion over the four-week treatment period. The higher dose of EU resulted in more substantial improvements in both parameters, suggesting enhanced recovery of neuromotor coordination and muscle function. The progressive reduction in foot splay distance and gait scores among EU-treated groups points to its neuroprotective potential, likely attributed to its antioxidant, anti-inflammatory, and neuromodulatory properties. These findings underscore the therapeutic promise of EU in counteracting ACR-induced behavioral and motor impairments, as illustrated in Fig. 1.

### Effect of eugenol on motor coordination (fall latency) in the rotarod test in ACR-induced brain toxicity in rats

**Fig. 2:**
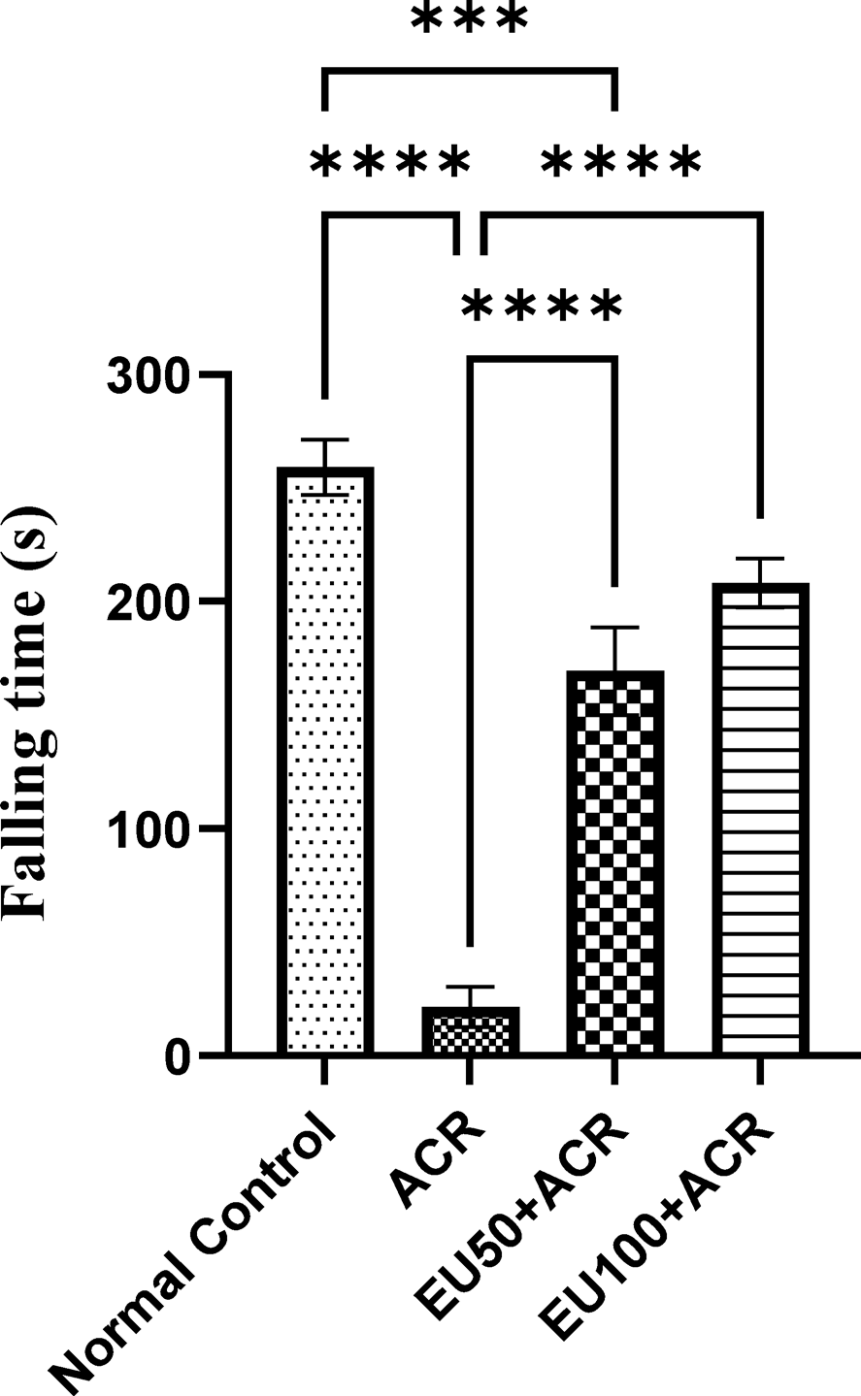
Effect of eugenol on falling time in the rotarod test in rats with ACR-induced brain toxicity. The data are presented as mean ± SEM (n = 6). “***, ****” represents a *P* value < 0.0001.

ACR administration significantly impaired motor coordination in rats, as evidenced by a marked reduction in fall latency during the rotarod test compared to the normal control group. This decline highlights the motor deficits associated with ACR-induced neurotoxicity. However, treatment with EU at doses of 50 and 100 mg/kg markedly improved motor performance, as indicated by prolonged falling times. The improvement was dose-dependent, with the higher dose (EU100) showing a more pronounced effect. These findings suggest that EU effectively counteracts ACR-induced motor impairments, possibly through its neuroprotective and antioxidant properties, as demonstrated in Fig. 2.

### Effect of eugenol on brain oxidative/nitrosative stress markers in ACR-induced brain toxicity in rats

**Fig. 3:**
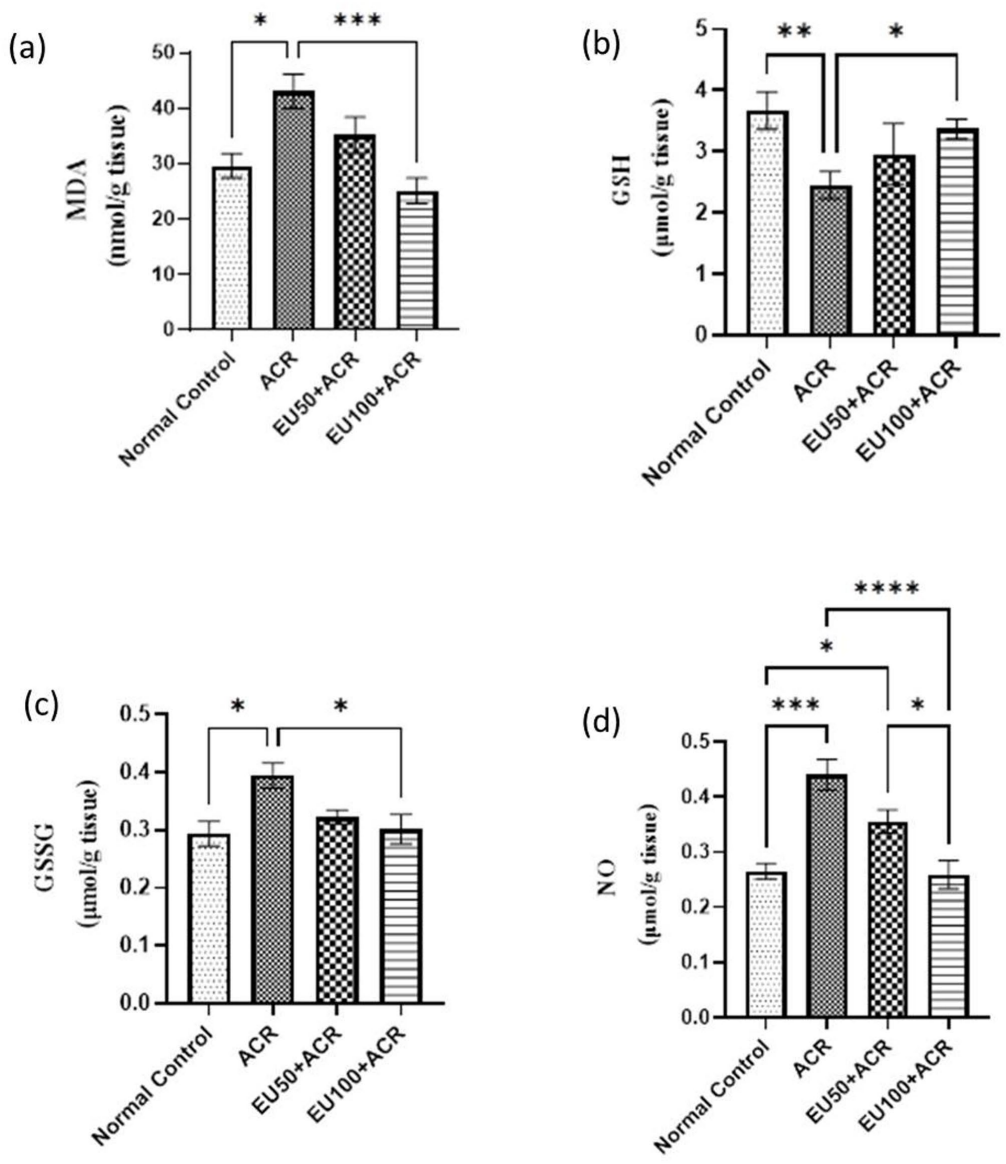
Effects of eugenol on brain oxidative/nitrosative stress biomarkers evidenced by MDA (**a**)**,** GSH (**b**), GSSG (**c**), and NO (**d**) in rats with ACR-induced brain toxicity. The data are presented as mean ± SEM (n = 6). “*” represents a *P* value < 0.05, “**” represents a *P* value < 0.001, and “***, ****” represents a *P* value < 0.0001.

As shown in Fig. 3, ACR administration caused a significant disruption in redox homeostasis, as reflected by elevated levels of MDA (a) and NO (d), along with a reduction in key antioxidant defense components, including GSH (b) and an increase in GSSG (c).

Specifically, ACR exposure led to a significant increase in MDA levels, a well-established marker of lipid peroxidation, suggesting enhanced membrane oxidative damage. Simultaneously, NO levels were significantly elevated, reflecting excessive nitrosative stress that can exacerbate neural inflammation and cellular injury. These biochemical alterations were accompanied by a depletion of GSH and an increase in GSSG, indicating compromised cellular antioxidant capacity and redox imbalance.

Conversely, treatment with EU, particularly at the higher dose (100 mg/kg), markedly restored redox balance. MDA and NO levels were significantly reduced in EU-treated groups, while GSH levels increased and GSSG levels declined, demonstrating the potent antioxidant and free radical-scavenging capacity of EU. These effects were dose-dependent, with EU100 showing a more pronounced protective effect than EU50.

Overall, these findings highlight the efficacy of EU in mitigating ACR-induced oxidative and nitrosative stress, thereby contributing to its neuroprotective potential in the context of brain toxicity.

### Effect of eugenol on brain Nrf2/HO-1/NQO1 antioxidant signaling pathway in ACR-induced brain toxicity in rats

**Fig. 4:**
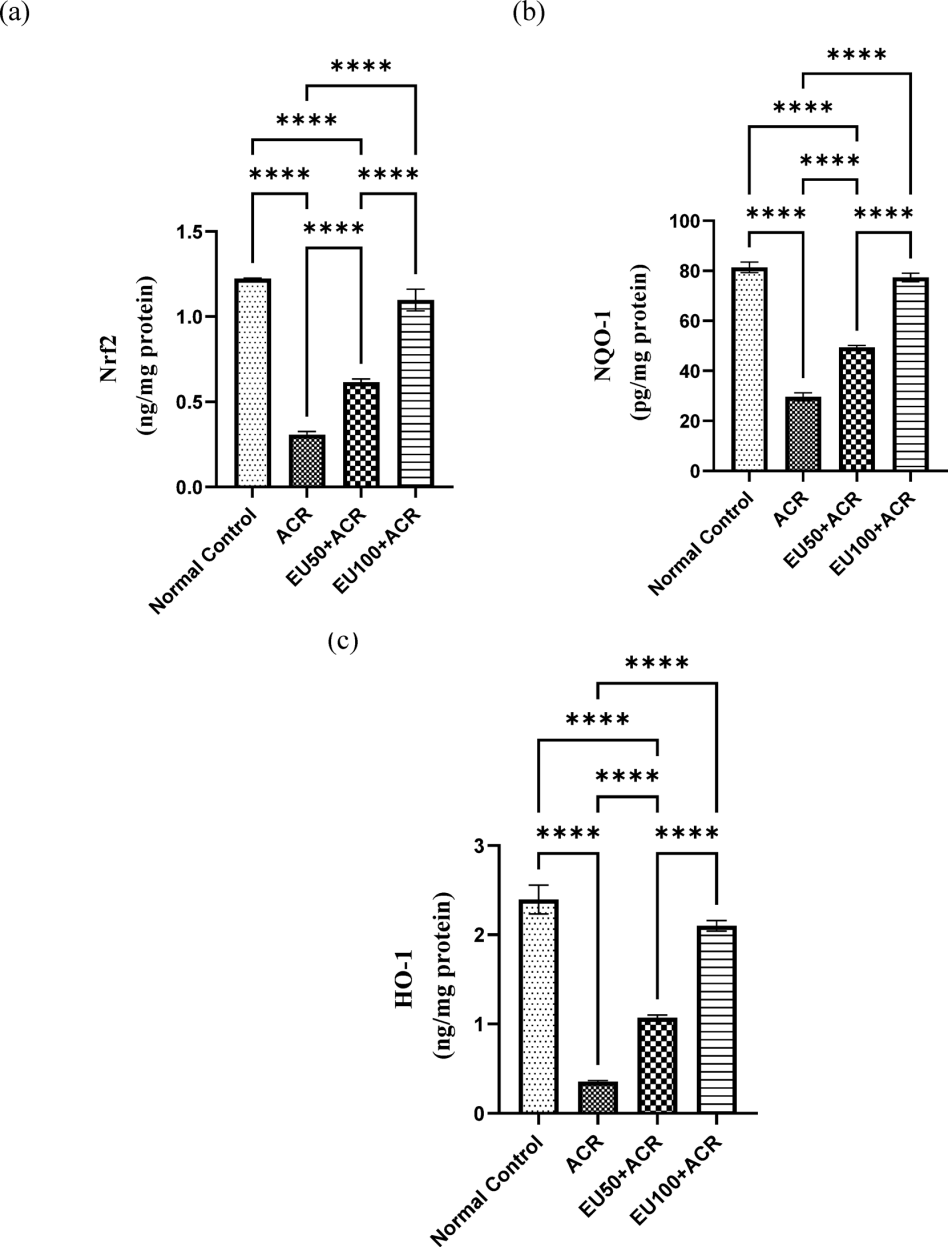
Effects of eugenol on brain Nrf2/NQO1/HO-1 in rats with ACR-induced brain toxicity. The data are presented as mean ± SEM (n = 6). “****” represents a *P* value < 0.001.

As demonstrated in Fig. 4, ACR administration significantly downregulated the expression of Nrf2 (a), NQO1 (b), and HO-1(c), reflecting impaired activation of the endogenous antioxidant response system. This suppression of the Nrf2 signaling axis suggests a mechanistic link between ACR-induced oxidative injury and inadequate cytoprotective responses in brain tissues. Notably, treatment with EU, especially at 100 mg/kg, markedly restored the expression of these antioxidant markers. Nrf2 levels were significantly elevated in both EU-treated groups compared to the ACR group, indicating reactivation of the master transcription factor responsible for antioxidant defense. Correspondingly, the downstream effectors NQO1 and HO-1 also showed significant upregulation following EU administration, with the higher dose yielding near-normalization of protein levels.

These findings suggest that the neuroprotective effects of EU against ACR-induced toxicity are mediated, at least in part, through activation of the Nrf2/NQO1/HO-1 signaling pathway. This activation enhances the cellular antioxidant capacity, mitigates oxidative damage, and contributes to restoring redox homeostasis, thereby protecting neuronal and reproductive function under ACR-induced stress conditions.

### Effect of eugenol on brain NLRP3 inflammasome/NF-κB/IL-1β signaling pathway in ACR-induced brain toxicity in rats

**Fig. 5:**
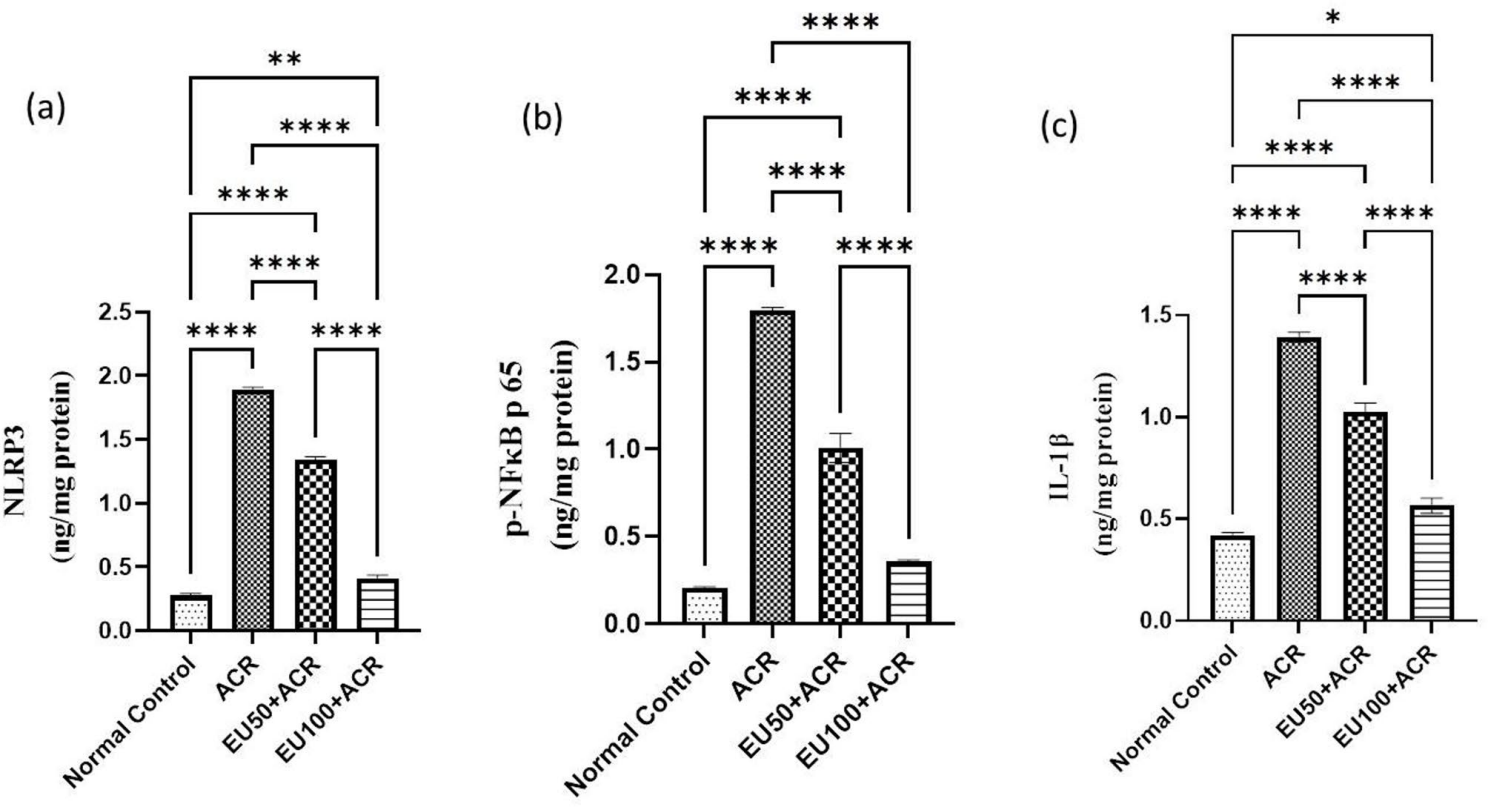
Effects of eugenol on brain NLRP3/NF-κB/IL-1β in rats with ACR-induced brain toxicity. The data are presented as mean ± SEM (n = 6). “*” represents a *P* value < 0.05, “**” represents a *P* value < 0.001, and “****” represents a *P* value < 0.0001.

Inflammation plays a central role in ACR-induced neuroendocrine damage, and the NLRP3 inflammasome is a key upstream mediator of this response. As shown in Fig. 5, ACR administration led to a marked activation of the NLRP3 inflammasome, reflected by a significant increase in NLRP3 protein levels (a). This was accompanied by elevated expression of phosphorylated NF-κB p65 (b), a master transcription factor for pro-inflammatory cytokines, and a substantial rise in the downstream effector cytokine IL-1β (c).

These findings highlight the proinflammatory environment triggered by ACR toxicity, which likely contributes to the observed behavioral, biochemical, and histological impairments. Notably, treatment with EU significantly attenuated these inflammatory responses in a dose-dependent manner. Both EU50 and EU100 reduced the expression of NLRP3, p-NF-κB, and IL-1β, with the higher dose showing a more pronounced inhibitory effect. The suppression of this signaling axis suggests that EU may interrupt the upstream activation of the inflammasome, inhibit NF-κB nuclear translocation, and consequently limit the release of pro-inflammatory cytokines.

### Effect of eugenol on the brain histopathological findings in ACR-induced brain toxicity in rats

The number of degenerate neurons in the cerebral cortex and hippocampus as well as the number of degenerate Purkinje cells is illustrated in Table 1.

**Table 1:** demonstrates the number of degenerate neurons in the cerebral cortex and hippocampus as well as the number of degenerate Purkinje cells in the different groups.

Groups	The numbers of degenerate neurons (mean ± SE)		
	The cerebral cortex	The hippocampus	Purkinje cells (cerebellum)
Control	2.00^c^ ± 0.31	0.20^c ^± 0.20	1.20^b^ ± 0.20
ACR	21.40^a^ ± 2.11	11.00^a ^± 1.30	9.60^a^ ± 1.70
EU50 + ACR	7.60^b ^± 0.67	4.20^b^ ± 0.73	1.60^b ^± 0.40
EU100 + ACR	5.40^b^,^c^ ± 0.67	1.40^c ^± 0.24	1.20^b ^± 0.48

Values expressed as mean ± SEM (n = 5 random fields). Values with dissimilar letters means significantly different at *p* < 0.5

**Fig. 6:**
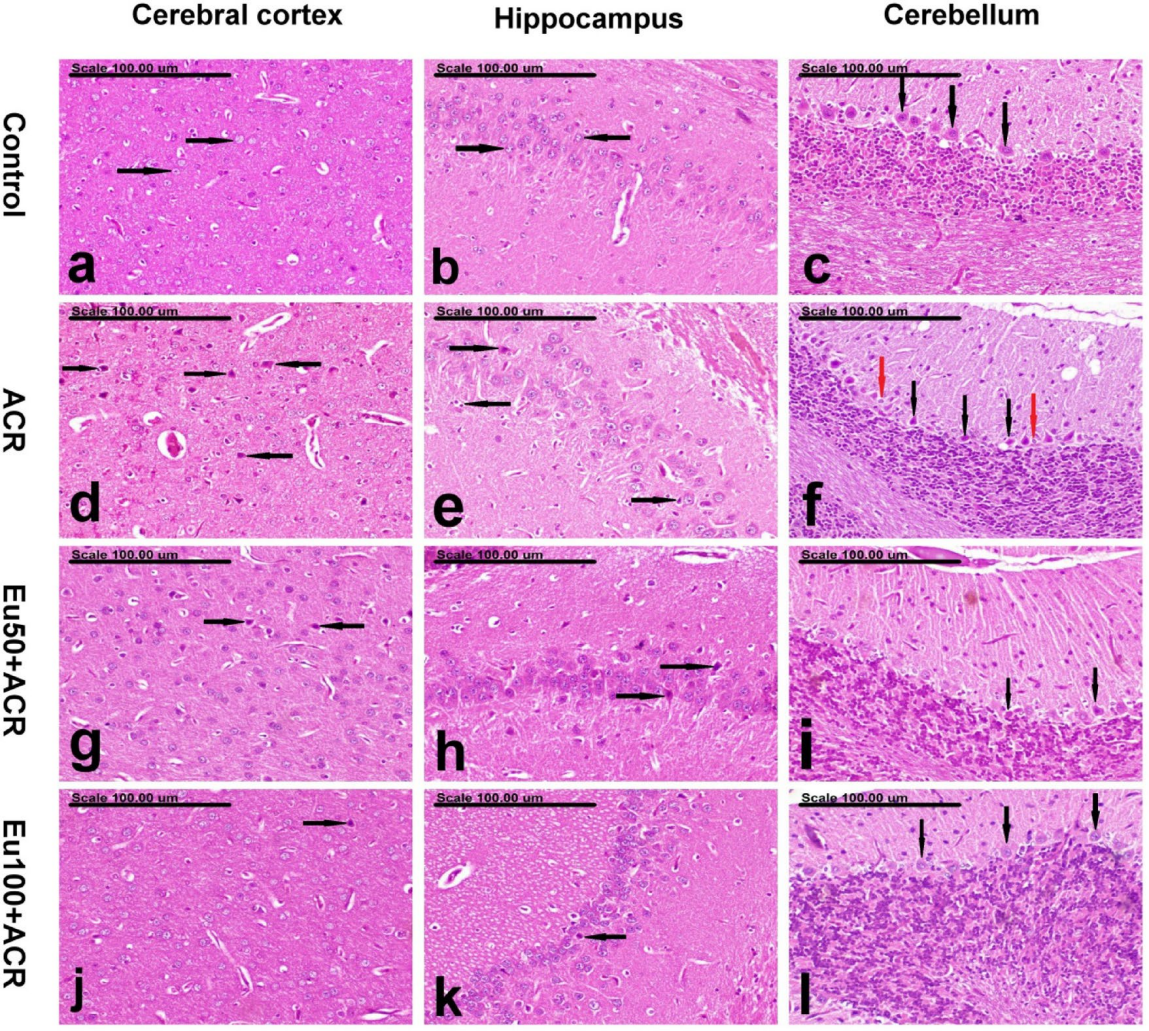
Effect of eugenol on the brain histopathological findings in ACR-induced brain toxicity in rats. Photomicrograph showing the brains of (**a**, **b**, and **c**) the control group, showing normal cerebral cortex with normal round neurons (black arrows) (**a**), normal hippocampal neurons (black arrows) (**b**), and normal Purkinje cells (black arrows) at the interface of the outer molecular and inner granular cell layers (**c**), (**d**, **e**, **f**) ACR group showing an increased number of degenerated neurons with intensely eosinophilic cytoplasm and pyknotic nuclei in the cerebral cortex (black arrows) (**d**), neuronal degeneration with neuronophagia in the hippocampus (black arrows) (**e**), and degeneration of the Purkinje cells (black arrows), associated with proliferation of astrocytes (red arrows) (**f**), (**g**, **h**, **i**) Eu50 + ACR group showing a significant reduction in the number of degenerating neurons in the cerebral cortex (black arrows) (**g**) and hippocampus (black arrows) (**g**), in addition to a decreased number of degenerate Purkinje cells (black arrows) (**i**) and (**j**, **k**, **l**) Eu100 + ACR group showing sparse degenerate neurons in the cerebral cortex (black arrow) (**j**) and hippocampus (black arrow) (**k**) and normal Purkinje cells (black arrows) (**l**). (Stain: H&E, scale bar = 100 µm).

The brains of the control group revealed normal cerebral cortex and hippocampus (Figs. 6a and b). The cerebellum of this group showed normal Purkinje cells at the interface of the outer molecular and inner granular cell layers (Fig. 6c). On the contrary, the cerebral cortex of the ACR group revealed wide-spread neuronal degeneration with an increased number of degenerated neurons. The degenerated neurons exhibited intensely eosinophilic cytoplasm and pyknotic nuclei (Fig. 6d). The hippocampus of this group revealed neuronal degeneration with neuronophagia, which is characteristically demonstrated in the CA1 and CA2 subfields (Fig. 6e). Additionally, degeneration of the Purkinje cells, associated with proliferation of astrocytes, was demonstrated in the cerebellum of this group (Fig. 6f). Pronounced improvement was demonstrated in the brains of the Eu50 + ACR group, with a significant reduction in the number of degenerating neurons in the cerebral cortex and hippocampus (Figs. 6g and h). The number of degenerate Purkinje cells was also reduced in this group (Fig. 6i). Compared to the Eu50 + ACR group, better improvement was demonstrated in the brains of the Eu100 + ACR group, which revealed sparse degenerate neurons in the cerebral cortex and hippocampus (Figs. 6j and k).

### Effect of eugenol on brain caspase-3 immunohistochemical expression in ACR-induced brain toxicity in rats

**Fig. 7:**
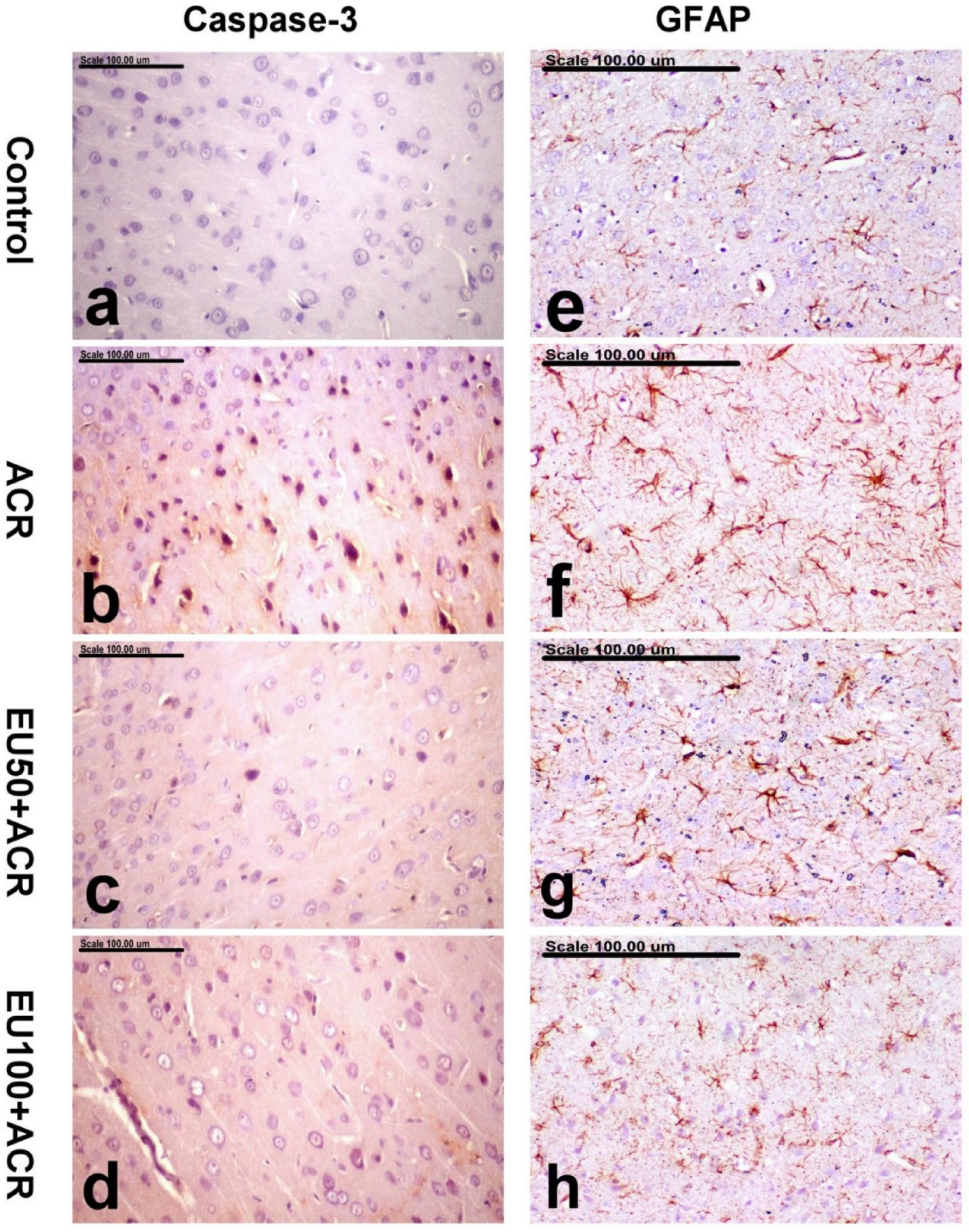
Effects of eugenol on brain caspase-3 and GFAP immunohistochemical expressions in ACR-induced brain toxicity in rats. Photomicrograph showing immunohistochemically stained cerebral cortex of (**a**, **e**) the control group showing no caspase-3-positive cells (**a**) and normal GFAP + astrocytes with few, small processes (**e**), (**b**, **f**) ACR group showing a significant increase in the percentage of caspase-3-positive cells with strong brown perinuclear and/or nuclear staining (**b**) and numerous GFAP + astrocytes with hypertrophied cell bodies and thickened arborizing processes (**f**), (**c**, **g**) Eu50 + ACR group showing a significantly decreased percentage of caspase-3-positive cells (**c**) and a significant decrease in the number of hypertrophied GFAP + astrocytes (**g**), (**d**, **h**) Eu100 + ACR group showing individual caspase-3-positive cells (**d**) and a significant decrease of hypertrophied GFAP + astrocytes (**h**). (Stain: anti-Caspase-3 (**a**, **b**, **c**, **d**) and anti-GFAP (**e**, **f**, **g**, **h**), scale bar = 100 µm).

Immunohistochemical analysis of caspase-3 expression in brain sections revealed marked differences among the experimental groups, as summarized in Table 2. In the normal control group, no caspase-3-positive cells were detected in the cerebral cortex, indicating the absence of apoptotic activity (Fig. 7a). In contrast, rats exposed to ACR exhibited a significant increase in caspase-3-positive cells, characterized by intense brown perinuclear and/or nuclear staining (Fig. 7b) reflecting extensive apoptotic neurodegeneration.

**Table 2:** demonstrates the results of caspase-3 expression and GFAP^+^cells in the cerebral cortex of the control and other treated groups.

Groups	Casapase-3 expression (% of positive cells/HPF)	GFAP^+^cells (No./um)
Control	0.10^d^ ± 0.10	0.10^d^ ± 0.10
ACR	3.30^a^ ± 0.21	3.30^a^ ± 0.21
EU50 + ACR	1.80^b^ ± 0.24	1.80^b^ ± 0.24
EU100 + ACR	1.00^c^ ± 0.14	1.00^c^ ± 0.14

Values expressed as mean ± SEM (n = 5 random fields). Values with dissimilar letters means significantly different at *p* < 0.5

Co-treatment with EU at both 50 mg/kg (EU50) and 100 mg/kg (EU100) markedly reduced the percentage of caspase-3-positive cells compared to the ACR group (Figs. 7c and d, respectively). The reduction was dose-dependent, with EU100 providing a more pronounced decrease in caspase-3 immunoreactivity than EU50. These findings suggest that EU effectively mitigates ACR-induced neuronal apoptosis, likely through its antioxidant and anti-inflammatory properties, thereby contributing to its neuroprotective profile.

### Effect of eugenol on brain GFAP immunohistochemical expression in ACR-induced brain toxicity in rats

The immunostaining of glial fibrillary acidic protein (GFAP), a hallmark marker of astrocyte activation, is presented in Table 2 and Fig. 7. In the control group, GFAP + astrocytes displayed normal morphology with small cell bodies and thin, short processes, consistent with a non-reactive glial state (Fig. 7e). However, in the ACR-treated group, there was a marked increase in the number and reactivity of GFAP + astrocytes, evident by hypertrophied cell bodies and thickened, arborizing processes (Fig. 7f), indicating reactive astrogliosis in response to neurotoxic insult.

Treatment with EU at both 50 mg/kg and 100 mg/kg significantly attenuated this astrocytic response. The number of hypertrophied GFAP + astrocytes was considerably reduced in the cerebral cortex of EU50 + ACR and EU100 + ACR groups (Fig. 7g and h, respectively), with the higher dose yielding a more substantial normalization of astrocyte morphology and density. These results imply that EU suppresses ACR-induced glial activation and neuroinflammation, further supporting its neuroprotective potential.

## Discussion

In humans and animals, ACR exerts a neurotoxic effect on the nervous system, potentially leading to various disorders, including neurotoxic syndrome characterized by specific features such as skeletal muscle weakness and ataxia. Prior research has recorded the neurotoxic impacts of ACR in work environments with clinical manifestations comprising ataxia, foot splay, and skeletal muscle weakness^26^. Daily exposure to ACR results in the manifestation of neurological symptoms in different lab animals that are akin to those seen in humans with ACR-induced neurotoxicity^27^. In the present work, an obvious motor impairment has been developed following ACR (20 mg/kg/day) exposure in rats after 7 days, with muscle weakness, ataxia, and gait instability that get worsen on weekly basis for 4 weeks. Additionally, it has been demonstrated that the administration of ACR harms cellular macromolecules and leads to neuronal degeneration, both of which are primary contributors to motor impairment^28^. It has been established that ataxia arises from dysfunctional limb movement, which is most often due to cerebellar dysfunction impacting primarily Purkinje neurons and a few granule neurons^29^. Our investigation also revealed an apparent loss of Purkinje cells in the cerebellum, astrocytosis, and apoptosis in rats subjected to ACR that might explained the observed motor disability. These outcomes are in line with those obtained from our previous work^30^.

On the contrary, oral gavage of EU in a dose-dependent manner improved the motor disability associated with ACR exposure in rats, as verified by the noticeable improvement in gait score, and landing foot splay distance, alongside successful preservation of motor coordination capability on weekly basis for 4 consecutive weeks. This favorable impact of EU against ACR-induced neurotoxicity could be explained by the observed restoration of the cerebellum’s Purkinje cells in the EU-treated groups.

Furthermore, ACR’s neurotoxic effects have been linked to oxidative stress^31^. The interaction of ACR and its metabolite; glycidamide to GSH may contribute to the loss of GSH content subsequently MDA elevation in rats treated with ACR, resulting in oxidative stress^32^. Decidedly, the activation of the Nrf2 signaling pathway serves as a crucial compensated protective mechanism to reduce oxidative damage caused by ACR. When Nrf2 is activated, it rapidly separates from Keap1 and then moves into the nucleus, where it attaches to the antioxidant response element and triggers the activation of phase II detoxifying enzymes such as GSH^33^. The proteins HO-1 and NQO-1 serve as significant downstream target genes within the Nrf2 pathway^34^. HO-1 activity has a direct impact on cells’ capacity to counter oxidative damage, while NQO-1 is crucial for processes such as cell transformation, apoptosis, and cellular protection. Our findings corroborated the presence of oxidative stress in the brain tissues of rats treated with ACR, as evidenced by an elevation in brain MDA, NO, and GSSG levels, coupled with significant reduction in nuclear Nrf2, HO-1, NQO1, and non-enzymatic antioxidant GSH content, which are in accordance with former research^35,36^. Conversely, EU co-treatment with ACR markedly alleviated oxidative conditions in brain tissue, as demonstrated by significant up-regulation in the levels of Nrf2 and its downstream mediators; HO-1, NQO, and GSH with a subsequent decline in MDA, NO, and oxidized from of GSH. This outcome might be ascribed to the antioxidant capacity of EU as earlier validated^37^. Prior studies have demonstrated that EU can produce antioxidant effects by reducing intracellular levels of ROS, H_2_O_2_, and NO^38^. Chen et al. has also demonstrated a similar outcome, suggesting that EU alleviates colitis by activating the intestinal Keap1-Nrf2 signaling pathway to diminish oxidative stress^39^. Additionally, in a study on H_2_O_2_-induced damage in NIH-3T3 cells, EU markedly lessened oxidative response through up-regulating the expression level of Nrf2^37^. Moreover, previous investigation depicted the powerful antioxidant action of EU to reduce the oxidative/nitrosative indicators in ACR-induced testicular dysfunction in rats^16^.

Inflammasomes are intracellular multi-protein complexes, marked by the activation of inflammatory caspases^40^. Canonical inflammasomes consist of a ligand-sensing receptor, comprising members of the NLR family like NLRP3. Nevertheless, the uncontrolled activation of inflammasomes plays a part in different autoimmune and inflammatory conditions^41^. Due to the importance of the NLRP3 inflammasome in human central nervous system disorders, studies have been conducted on the potential for pharmacological targeting the NLRP3 signaling pathways. Research has demonstrated that serious neurodegenerative disorders (like cerebellar ataxias) manifest early and irreversible processes, with considerable microgliosis induced by the NLRP3 inflammasome pathway in the cerebellum. In contrast, inhibiting NLRP3 function in vivo markedly postpones neuronal degeneration and the onset of ataxia^42^. At present, there have been multiple advancements in pinpointing exogenous substances that can inhibit the activation of the NLRP3 inflammasome^43,44^.

NF-κB, an inducible transcription factor found in neurons and neuroglia, has been associated with inflammation triggered by various stimuli^45^. The involvement of the NF-κB signaling pathway in the regulation of the inflammasome, and onset and progression of inflammatory diseases, is now evident. NF-κB plays a crucial role in the priming signal for NLRP3 inflammasome activation, working by inducing the transcription of NLRP3 and pro-IL-1β in response to different ligands and cytokines^45^. Similar to the pro-IL-1β gene, the NLRP3 is a direct target of NF-κB and has NF-κB-binding sites located in its promoter region^46^. 65 Secretion of the mature and active forms of the cytokine IL-1β drives inflammatory responses via diverse downstream signaling pathways, leading to neuronal damage. IL-1β operates as a powerful cytokine that triggers the production of additional cytokines such as IL-6 and TNF-α, which exacerbates inflammation^47^. Consequently, the NLRP3 inflammasome is regarded as a major factor in the onset of neuroinflammation. Research has shown that the activation of the NLRP3 inflammasome plays a role in various neurological disorders, including brain infection, acute brain injury, and neurodegenerative diseases^48^. Zong et al. demonstrated that ACR-induced the up-regulation of pro-inflammatory cytokines via the NLRP3 inflammasome pathway in the rat cerebral cortex after gavage administration at a dosage of 20 mg/kg/day for 5 weeks^49^.

Recent studies have provided further evidence that dysregulation of the Nrf2/NLRP3 signaling pathway contributes to brain damage^50,51^. Therefore, the current research examined how the NLRP3 inflammasome pathway and Nrf2 signaling pathway are involved in neurotoxicity induced by ACR. Oxidative stress and inflammation significantly contribute to the development of acute and chronic injuries induced by ACR^52^. Notably, the Nrf2 and the NLRP3 inflammasome are linked to stress and inflammatory conditions^53^. A physical crosstalk between the two pathways has been proposed mechanistically, and it has been demonstrated that Nrf2 co-localizes with the key adaptor protein (ASC specks) in inflammasomes^54^. Consequently, the Nrf2 and NLRP3 inflammasome pathways are linked at various levels, primarily in an opposing or mutually antagonistic pattern. According to the review of Hennig et al., the interaction between Nrf2 and inflammasomes has shown that Nrf2 activation can suppress inflammasomes and inflammatory response in various inflammation-related disease models^55^. According to previous research, the activation of Nrf2-antioxidant signaling can reduce NLRP3 inflammasome activity by inhibiting the ROS-NLRP3-IL-1β signalling axis^56^. Herein and similar to prior investigation^57^, ACR toxicity exhibited neuroinflammation in the brain tissue through upregulation of NLRP3/NF-κB/IL-1β pathway, explaining the observed decline in Nrf2 signaling pathway. Moreover, these outcomes indicate that the Nrf2/NLRP3 axis is a key mechanism underlying ACR-induced neuronal injury. Meanwhile, oral administration of EU dose-dependently significantly relieved neuroinflammation brought on by ACR through inhibiting the NLRP3/NF-κB/IL-1β axis, signifying its anti-inflammatory potential. Several studies emphasized the efficacy of EU in inhibiting inflammatory response through suppressing NF-κB and NLRP3 signaling pathways^58,59^. In addition, previous studies have concluded that EU or its analogues could stimulate Nrf2 nuclear translocation and consequently upregulate downstream antioxidant enzymes, thereby decreasing intracellular ROS accumulation and subsequent activation of the NLRP3 inflammasome^60,61^. This bidirectional regulation suggests that restoration of redox balance serves as a central mechanism by which EU could modulate both oxidative stress and inflammation pathways in such neurotoxic conditions.

Apoptosis could be a subsequent process initiated in cell under oxido-inflammatory conditions. Explicitly, NF-κB seems to be involved in implementation of apoptosis by provoking inflammation and changing the expression levels of pro-apoptotic markers like caspase-3. Caspase-3 is a key regulator of apoptosis^62^, which causes disruptions in the cytoskeleton, nucleus, and other cellular changes associated with apoptosis. Multiple ACR-induced neurological disorder models documented the role of oxidative insult and cytokine release on stimulating apoptosis in the brain. As stated by Abou Zaid et al., the oxidative stress resulting from ACR administration led to apoptosis and necrosis, an increase in degenerative plaques, and dilation of blood capillaries in the brain’s striatum and hippocampus^63^. According to Farouk et al., free radicals generated by ACR at elevated levels led to hemorrhaging and congestion in the blood vessels of rat brain tissue^64^. Additionally, in rats treated with ACR, low levels of antioxidant enzymes resulted in the degeneration of pyramidal and glial cells, as well as neuron necrosis^65^. In our study, an intense caspase-3 immunoexpression was detected in the cerebral cortex of ACR-control rats, strengthening the role of oxidative stress and inflammation in ACR-induced neuronal damage. By contrast, concurrent administration of EU to ACR-challenged rats dampened the apoptosis process in the brain tissue, as evidenced by the low percentage of caspase-3-postive cells at the high dose level. This outcome verified the anti-apoptotic potential of EU which could be highly ascribed to its antioxidant and ant-inflammatory properties.

Furthermore, astrocytes are also vital for preserving the brain’s structure, with GFAP serving as a key intermediate filament^66^. Thus, GFAP has been deduced to be a sensitive indicator for reactive astrogliosis consequently neuroinflammation^67–69^. In our study, numerous GFAP + astrocytes with hypertrophied cell bodies and thickened arborizing processes indicate brain injury and degeneration, as observed in rat brains exposed to ACR. These findings confirm earlier report, underlining the ACR-provoked astrocytosis^70^. Nevertheless, EU administration, which is effective in a dose-dependent manner, significantly reduced the number of hypertrophied GFAP + astrocytes in the brain, underscoring its potential as a neuroprotective agent.

Moreover, our research shows that there are considerable degenerative disorders in the brain of rats exposed to ACR, which aligns with earlier models of neurotoxicity induced by ACR^30,63–65^. Sections of the brains of ACR-intoxicated rats showed degeneration of neurons in the cerebral cortex and hippocampus regions, in particular, CA1 and CA2 subfields, as well as degeneration of the Purkinje cells, associated with proliferation of astrocytes, in the cerebellum. Nevertheless, EU treatment effectively thwarted the occurrence of these pathologies in a dose-dependent manner. It is noteworthy that EU at high dose (100 mg/kg) effectively prevented major degeneration and preserved normal Purkinje cells in the brain sections.

## Conclusion

Our investigation using a rat model demonstrated that ACR (20 mg/kg/4 weeks/orally)- poses considerable neurological threats, including hind limb paralysis, muscle weakness, and compromised locomotor activity, as well as evidence of oxidative damage, inflammation, and apoptosis in the brain tissue. Oral administration of EU (50 and 100 mg/kg), a potent anti-inflammatory and antioxidant agent, alleviated oxidative stress, restored levels of antioxidant enzymes, and reduced neuroinflammation through the activation of the Nrf2/NQO1 and HO1 pathway while down-regulating the NLRP3/NF-κB/IL-1β signaling. Furthermore, EU prevented neural apoptosis by suppressing caspase-3 expression and maintaining neuronal integrity. From all these results, we can conclude that EU is a potential neuroprotective agent against ACR-induced neurotoxicity and its importance in therapeutic strategies aimed at reducing neurotoxic damage is emphasized. Nevertheless, investigations incorporating western blot or immunofluorescence approaches to provide protein localization and activation patterns of Nrf2 and NLRP3 at the cellular level could be a scope of future studies.

## Data Availability

Data is provided within the manuscript.
